# Interacting Effects of Heat and Nanoplastics Affect Wheat (*Triticum turgidum* L.) Seedling Growth and Physiology

**DOI:** 10.3390/plants14152426

**Published:** 2025-08-05

**Authors:** Debora Fontanini, Stefania Bottega, Monica Ruffini Castiglione, Carmelina Spanò

**Affiliations:** 1 Department of Biology, University of Pisa, Via L. Ghini 13, 56126 Pisa, Italy; debora.fontanini@unipi.it (D.F.); stefania.bottega@unipi.it (S.B.); carmelina.spano@unipi.it (C.S.); 2 Center for Climate Change Impact, University of Pisa, Via Del Borghetto 80, 56126 Pisa, Italy

**Keywords:** polystyrene nanoparticles, high temperatures, oxidative stress, antioxidant response, *Triticum turgidum*, histochemical analysis, climate change

## Abstract

Nano- and microplastic pollution, together with the ongoing rise in global temperatures driven by climate change, represent increasingly critical environmental challenges. Although these stressors often co-occur in the environment, their combined effects on plant systems remain largely unexplored. To test the hypothesis that their interaction may exacerbate the effects observed under each stressor individually, we investigated the response of seedlings of *Triticum turgidum* to treatments with fluorescent polystyrene nanoplastics under optimal (25 °C) and elevated (35 °C) temperature conditions. We evaluated seedling growth, photosynthetic pigment content, and oxidative stress markers using both biochemical and histochemical techniques. In addition, we assessed enzymatic and non-enzymatic antioxidant responses. The use of fluorescently labeled nanoplastics enabled the visualization of their uptake and translocation within plant tissues. Elevated temperatures negatively affect plant growth, increasing the production of proline, a key protective molecule, and weakly activating secondary defense mechanisms. Nanoplastics disturbed wheat seedling physiology, with these effects being amplified under high temperature conditions. Combined stress enhances nanoplastic uptake in roots, increases oxidative damage, and alters antioxidant responses, reducing defense capacity in leaves while triggering compensatory mechanisms in roots. These findings underscore a concerning interaction between plastic pollution and climate warming in crop plants.

## 1. Introduction

Plastic has been widely used worldwide since its introduction in the 1950s. Its widespread use across various industries and its pervasive presence in everyday life products has led to our era being referred to as the ‘Plastic Age’ [1].

The extensive use of plastics, combined with inadequate disposal and recycling practices, is leading to the generation of vast amounts of waste, posing a risk of widespread contamination across environmental compartments. A recent paper presented a detailed projection study on the future of global plastic production and waste generation. Despite anticipated improvements in recycling practices, the study predicts that plastic waste generation will reach between 832.8 and 873.7 million tons by 2050 [2].

Plastics are largely non-biodegradable, and when exposed to physical and chemical factors in the environment, they gradually fragment into smaller particles, ultimately forming microplastics (particles < 5 mm) and nanoplastics (particles < 100 nm) [3].

These small-sized plastics, classified as secondary, along with primary micro- and nanoplastics, intentionally manufactured at the micro- or nanoscale, are ubiquitous across all environments, including aquatic, atmospheric, and terrestrial matrices, and are increasingly recognized as emerging pollutants [4].

Micro- and nanoplastics pose substantial ecotoxicological concerns, as they can cause both direct and indirect adverse effects on living organisms. Their inherently high surface-to-volume ratio significantly enhances their capacity to adsorb, concentrate, and transport co-contaminants and environmental pollutants. Furthermore, the nanoscale dimensions of the smallest particles facilitate their translocation across biological barriers, promote bioaccumulation within tissues, and enable intracellular localization, where they may disrupt essential cellular functions and biochemical pathways, especially when combined with other environmental stressors [4]. Among environmental stress factors, the ongoing temperature increase driven by climate change constitutes a further cause for serious alarm. Copernicus Climate Change Service (http://www.copernicus.eu/it/servizi/cambiamenti-climatici, accessed on 1 August 2025) reports concerning statistics on the global temperature increase in 2024, noting an average temperature of 15.10 °C, 0.12 °C higher than in 2023 and 1.6 °C above the pre-industrial baseline.

Although the toxicity of nanoplastics to plants depends on factors such as their type, size, concentration, and the specific plant species, the literature frequently reports negative effects of these particles on plant metabolism and growth. The observed effects include inhibition of germination and growth, disruptions in water and nutrient uptake, reduced photosynthetic performance, increased oxidative stress markers, altered antioxidant responses, and genotoxicity [5]. On the other hand, the detrimental effects of high temperatures on plant development and productivity are well documented [6]. Despite growing concern about the potential impact of nanoplastic pollution and temperature increases associated with climate change on living organisms, the combined effects of these stressors on plant health have rarely been addressed. In *Daphnia magna*, the combined exposure to polystyrene nanoparticles and elevated temperatures resulted in severe oxidative stress, which adversely affected reproductive performance [7]. Similarly, in zebrafish, a 1 °C temperature increase in conjunction with nanoplastic exposure disrupted circadian rhythms, caused brain damage, and led to significant alterations in the levels of several metabolites across multiple metabolic pathways [8]. Differently, in the photoautotrophic organism *Scenedesmus obliquus*, elevated temperatures reduced the toxicity of nanopolystyrene, modulating the activity of ROS-related enzymes [9].

To specifically investigate the potential interactive effects of nanoplastics and elevated temperatures in plants, we previously examined the response of the fern *Azolla filiculoides* Lam. exposed to polystyrene nanoplastics (NPs) at an optimal temperature (25 °C) or at an elevated temperature (35 °C) [3]. Both NPs and elevated temperatures adversely impacted the development and physiology of *A. filiculoides*, with histological, morphological, and photosynthetic analyses revealing an exacerbation of symptoms under combined stress conditions. The worsening of the nanoplastic effects under high-temperature conditions was consistent with the observed increase in NPs uptake at 35 °C.

A microcosm experiment examining the effects of temperature fluctuations on soft wheat seedlings exposed to polystyrene nanoplastics revealed a complex interaction between climate change and nanoplastic pollution, affecting both plant growth and reactive oxygen species (ROS) accumulation and metabolism [10]. Based on these findings, the authors highlight the potential threat that nanoplastic contamination poses to crop productivity under changing climatic conditions. A comprehensive understanding of the mechanisms governing plant responses to these combined stressors is essential for accurately evaluating ecological risks and developing targeted mitigation strategies. The limited research on this topic represents a significant knowledge gap that warrants urgent attention, especially in relation to the potential combined effects of these two stressors on food crops. Driven by these findings, we investigated the combined impact of nanoplastics exposure and elevated temperatures on *Triticum turgidum* L. subsp. *durum* (Desf.) Husn., a plant species of recognized agricultural and nutritional importance, under the assumption that high temperatures may exacerbate the negative effects of nanoplastics even in a terrestrial, food-intensive crop. To test this hypothesis, we evaluated the growth and the photosynthetic pigments content in wheat seedlings treated with fluorescent polystyrene nanoplastics at either 25 °C or 35 °C. Oxidative stress parameters were also assessed using both biochemical and histochemical methods, along with the plant’s enzymatic and non-enzymatic antioxidant responses.

Our selection of nanoplastics concentration (50 mg L^−1^) was based on concentration ranges commonly used in hydroponic studies, which typically span from 10 to 100 mg L^−1^ [11,12]. These experimental ranges were considerably higher than environmentally relevant concentration gradients, which are generally in the microgram per liter (µg L^−1^) to low milligram per liter (mg L^−1^) range [13], with localized hotspots, such as wastewater effluents or agricultural runoff areas, occasionally exhibiting elevated levels. These environmental concentration gradients reflect both spatial variability and current limitations in sampling and nanoscale detection technologies [13].

In this context, the concentration used in our hydroponic system exceeded most environmental measurements but was intentionally selected within a worst-case scenario framework. This strategy enabled us to deeply explore potential plant–nanoplastic interactions and likely implications for food chain transfer.

The nanoplastic particles used had a nominal diameter of 30 nm, ranging from 20 to 50 nm, as previously characterized by transmission electron microscopy [14]. This size range was selected based on earlier studies [15], demonstrating that nanoparticles of similar dimensions are capable of being taken up by plant tissues. The use of fluorescent nanoplastics enabled us to investigate their uptake and potential translocation within the plant.

## 2. Results

### 2.1. Effect of Heat and Nanoplastics on Plant Growth Parameters and Photosynthetic Pigments Content

In our experiments, the high temperature led to a decreased length of both roots and leaves (Table 1 and Table 2). The presence of NPs increased growth compared to the respective control, but the difference between C 35 °C and NP 35 °C was not significant in roots. Both elevated temperature and NPs exposure significantly influenced the measured growth parameters, as shown in Table 1, Table 2 and Table S1.

High temperature had a significant effect on photosynthetic pigments, inducing a decrease both in total chlorophyll and in carotenoid contents (Table 3); no significant differences were observed at either temperature following the addition of nanoplastics. Heat treatment caused a decrease in the Chla/Chlb ratio and an increase in the Carotenoids/total Chl ratio, with the highest value recorded in the NP 35 °C treatment (Table 3).

### 2.2. Tracking Nanoplastics Uptake

**Figure 1:**
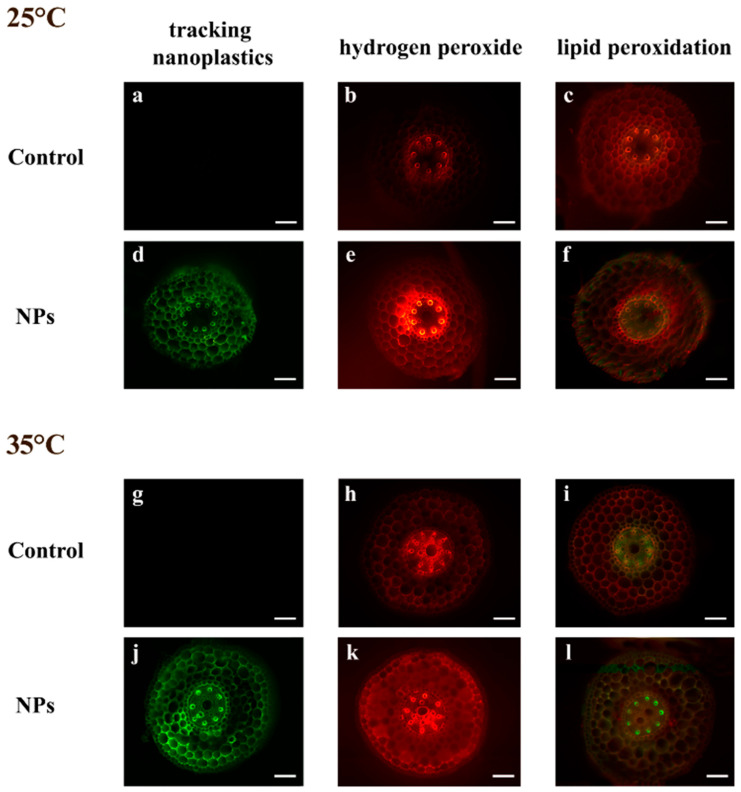
Cross hand sections of *T. turgidum* seedling roots after 7 days of imbibition. Seedlings were incubated in water at 25 °C (**a**–**c**) and 35 °C (**g**–**i**), and in the presence of polystyrene nanoparticles (NPs) at 25 °C (**d**–**f**) and 35 °C (**j**–**l**). Representative root sections are shown for green fluorescence of NPs (**a**,**d**,**g**,**j**); in situ detection of hydrogen peroxide using Amplex Red staining (**b**,**e**,**h**,**k**); and of lipid peroxidation using BODIPY staining (**c**,**f**,**i**,**l**). Scale bars = 150 μm.

**Figure 2:**
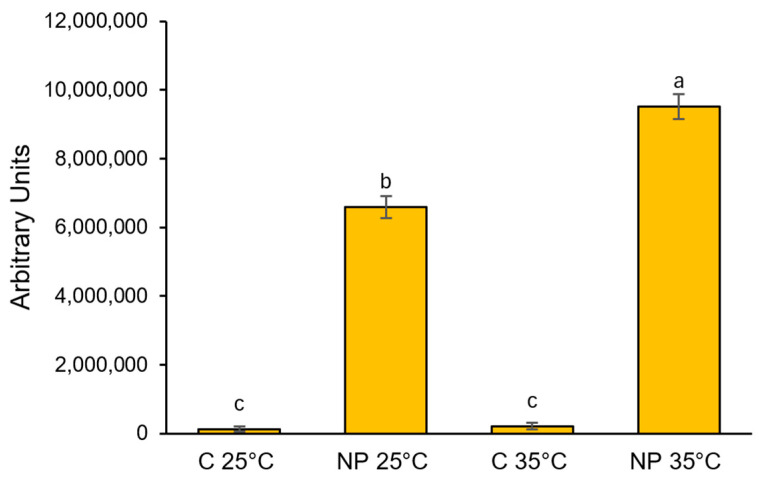
Polystyrene nanoparticles fluorescence intensity in root tissues of *T. turgidum* seedlings grown at 25 °C or 35 °C in water (C 25 °C, C 35 °C) or in the presence of polystyrene nanoplastics (NP 25 °C, NP 35 °C). Data are reported as mean ± standard error of at least four replicates. Different letters indicate statistically significant differences at *p* < 0.05.

The use of fluorophore-labeled nanopolystyrene allowed us to trace its distribution across different compartments of the root system in our model plant. In samples treated with nanoplastics at 25 °C, a diffuse green fluorescent signal was detected in the cortical cylinder, while in the central cylinder, fluorescence was limited to the xylem arches (Figure 1d). Following treatment at 35 °C, the green fluorescence intensified in both the cortex and the central cylinder, with the signal extending throughout the entire stele (Figure 1j). No fluorescent signal was observed in sections obtained from the control samples (Figure 1a,g). In addition, semi-quantitative analysis of fluorescence levels in root sections revealed that the uptake at root level increased by more than 40% at 35 °C compared to 25 °C (Figure 2).

### 2.3. Effect of Heat and Nanoplastics on Oxidative Stress Markers

**Figure 3:**
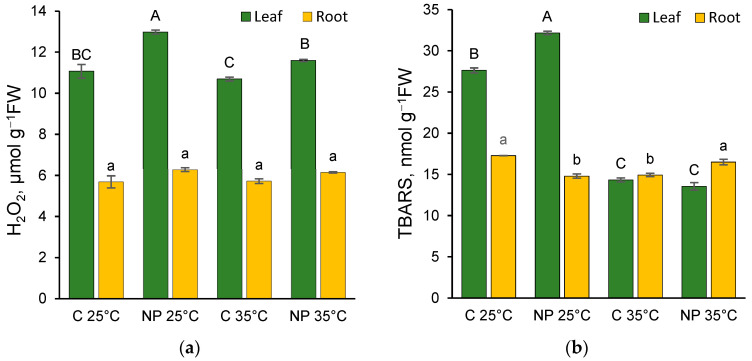
Hydrogen peroxide (**a**) and thiobarbituric acid reactive substances (TBARS) (**b**) content in *T. turgidum* seedling leaves and roots grown at 25 °C or 35 °C in water (C 25 °C, C 35 °C) or in the presence of polystyrene nanoplastics (NP 25 °C, NP 35 °C). Data are reported as mean ± standard error of at least four replicates. Different letters indicate statistically significant differences at *p* < 0.05, with uppercase letters referring to leaves and lowercase letters to roots.

In our experimental system, the presence of nanoplastics increased the hydrogen peroxide concentration in leaves at both temperatures, with the highest values observed at 25 °C (Figure 3a). The effect of both temperature and NPs, as well as their interaction, was significant (Table S2).

**Figure 4:**
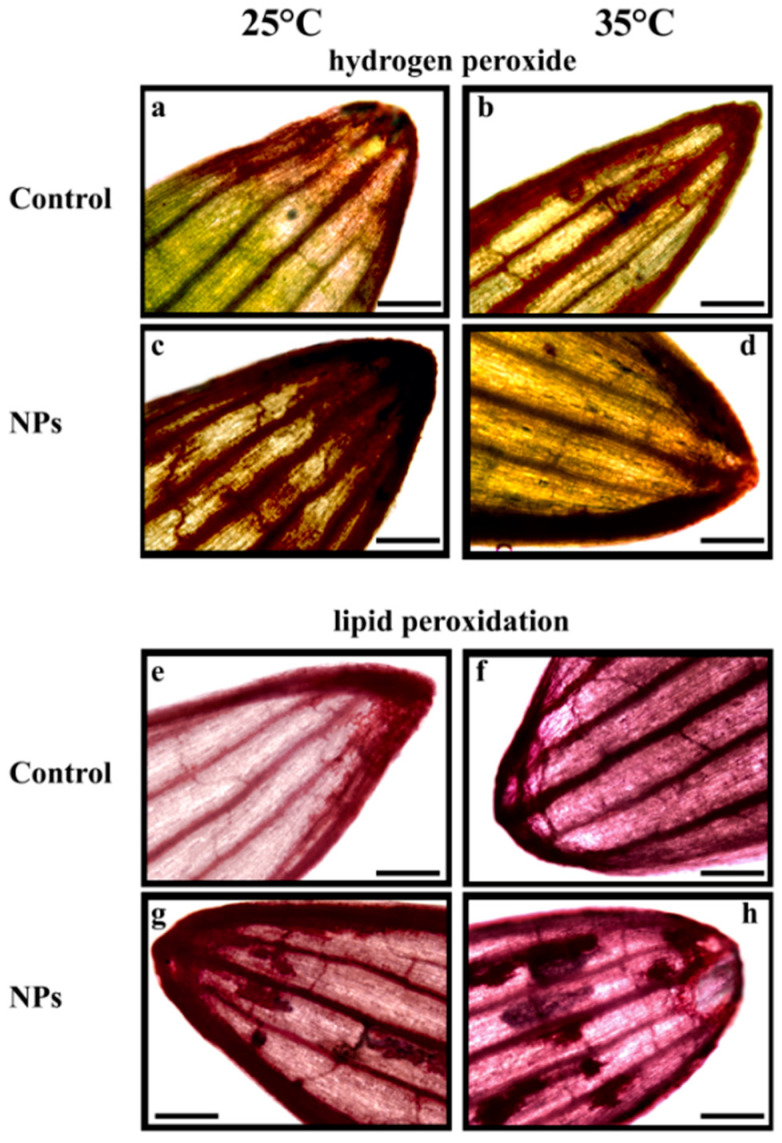
Leaf tips of *T. turgidum* seedling after 7 days of imbibition. Seedlings were incubated in water at 25 °C (**a**,**e**) and 35 °C (**b**,**f**), and in the presence of polystyrene nanoparticles (NPs) at 25 °C (**c**,**g**) and at 35 °C (**d**,**h**). Representative leaf tips are shown for histochemical detection of hydrogen peroxide using DAB staining (**a**–**d**) and of lipid peroxidation using Schiff’s reagent staining (**e**–**h**). Scale bars = 1 mm.

These findings were largely consistent with the histochemical analysis (Figure 4). In control leaves, DAB red-brown precipitates were predominantly localized at the leaf apex (Figure 4a). At 35 °C, however, the accumulation of brownish precipitates extended to the leaf margins and vascular tissues (Figure 4b). Leaves from the nanoplastic-treated samples showed a broader DAB reactivity (Figure 4c,d), particularly at 25 °C, where the staining pattern was more diffuse and pronounced (Figure 4c).

The level of hydrogen peroxide was significantly lower in the roots compared to the leaves, and no significant differences were observed across the various treatments (Figure 3a).

Histochemical analysis of the root apex revealed clear treatment-dependent differences, as illustrated in Figure 1. Exposure to plastics alone led to an increased reactivity of the Amplex probe compared to the control, with enhanced signal intensity observed in the central cylinder of the root, particularly at the xylem arches, as well as in the cortical region (Figure 1b,e). At 35 °C, the red staining pattern became more diffuse, extending across both the central cylinder and the cortex, with this effect being particularly pronounced in samples treated with nanoplastics (Figure 1h,k).

In leaves, TBARS, which indicate lipid peroxidation and, indirectly, membrane damage, showed higher values at 25 °C, with the highest content recorded in the NP 25 °C treatment (Figure 3b). There were no significant differences between C 35 °C and NP 35 °C. Both temperature and NPs appeared to have a significant effect, even in interaction with each other (Table S2). However, the high value of F and its significance suggest that temperature plays a predominant role.

Histochemical analysis also indicated that elevated temperatures induced significant oxidative damage, as shown by Schiff reagent staining (Figure 4e–h). Compared to the control, higher temperatures resulted in an increased purple coloration throughout the leaf lamina and along the veins (Figure 4e,f). Moreover, the presence of plastics at both 25 °C and 35 °C led to localized purple precipitates on the lamina, indicating severe oxidative damage (Figure 4g,h).

A different pattern was observed in the roots (Figure 3b), where nanoplastics led to a decrease in this oxidative damage marker at 25 °C and an increase at 35 °C. Although neither factor had a significant effect on its own, their interaction was significant (Table S4). In contrast, histochemical analysis of the root apex using the BODIPY probe revealed a distinct pattern of oxidative damage across different root tissues. At 25 °C, the presence of plastics in the culture medium induced a weak and fairly homogeneous green fluorescence in the root, indicative of mild oxidative damage (Figure 1c,f). In contrast, at 35 °C, the central cylinder exhibited heightened sensitivity to staining. The combined effect of elevated temperature and plastic exposure resulted in the most pronounced histochemically detectable oxidative damage, characterized by both diffuse green staining and intensely fluorescent regions, particularly in the xylem arches and cortical areas (Figure 1l).

### 2.4. Effect of Heat and Nanoplastics on Proline Level

The highest concentration of proline was observed in C 35 °C leaves, but the presence of NPs significantly reduced its concentration (Table 1). This parameter was significantly affected by both temperature and nanoplastics, as well as by their interaction (Table S2). The highest proline concentration in the roots was observed at 25 °C (Table 2), and the presence of NPs reduced its content at both temperatures, although the difference was significant only at 25 °C. Both temperature and NPs, as well as their interaction, had a significant impact on this parameter (Table S4).

### 2.5. Effect of Heat and Nanoplastics on Soluble Protein Content and Enzymatic Activity

The only significant difference in soluble protein content was observed in the roots at 35 °C, where the maximum protein concentration was recorded (Table 2). Temperature was the only factor that had a significant impact on this parameter (Table S5). Indeed, the presence of NPs led to a decrease in root protein content, but the effect was not statistically significant. Interaction between the two factors was found to be significant at *p* < 0.05 (Table S5). The highest APX activities in the leaves were always observed at 35 °C, with significantly lower values at 25 °C (Table 1). The addition of NPs increased the activity of this hydrogen peroxide-scavenging enzyme at 25 °C, while at 35 °C, it decreased the activity, though the levels remained higher than those recorded at 25 °C. The effect of temperature appeared to be predominant, with only a minor influence attributed to NPs. The interaction between the two factors was significant (Table S3). In the roots, only at 35 °C, the activity was significantly lower than in the other treatments, with a prevalent role played by NPs (Table 2). In this case as well, the interaction between the two factors was significant (Table S5). In the leaves, as observed for APX, POX activity increased at 35 °C, but the presence of NPs had an inhibitory effect (Table 1). Both factors had a significant effect, even when interacting with each other (Table S3). In the roots, POX activity remained relatively high compared to the leaves, with no significant differences observed among the different treatments (Table 2). In the leaves, both NPs and high temperatures had an inhibitory effect on CAT (Table S3), which reached its highest activity in C 25 °C; the addition of NPs led to a decrease in enzyme activity at both temperatures (Table 1). Although both factors had a significant effect, even in interaction, the presence of NPs appeared to play a predominant role, based on F values and significance (Table S3). In the roots, CAT activity was significantly higher in C 25 °C compared to the other treatments (Table 2).

### 2.6. Effect of Heat and Nanoplastics on Polyphenols Content

**Figure 5:**
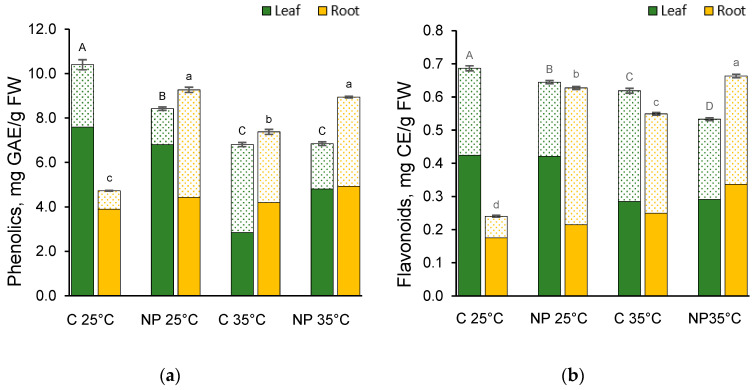
Phenolic compounds (**a**) and flavonoids (**b**) content in *T. turgidum* seedling leaves and roots grown at 25 °C or 35 °C in water (C 25 °C, C 35 °C) or in the presence of polystyrene nanoplastics (NP 25 °C, NP 35 °C). Each bar represents the sum of free (solid bars) and bound (dotted bars) metabolites. Data are reported as mean ± standard error of at least four replicates. Different letters indicate statistically significant differences at *p* < 0.05, with uppercase letters referring to leaves and lowercase letters to roots.

When wheat plants were grown at optimal temperature (25 °C) in the presence of NPs, a significant reduction was observed both in the leaves’ total phenol and flavonoid contents (Figure 5a,b). In contrast, the levels of phenolic compounds detected in leaves treated with NPs at 35 °C showed no difference from the C 35 °C, albeit being lower overall. On the other hand, under those conditions, the levels of flavonoids were significantly reduced with respect to the C 35 °C. At 35 °C, both controls and treated samples had lower content of total phenols and flavonoids compared to their 25 °C counterparts. Both temperature and NPs had significant effects, although the temperature factor had a prominent effect on phenolic compounds and NPs on flavonoids (Table S2).

In roots, total phenolics and flavonoids had similar content distribution (Figure 5a,b) with higher amounts of metabolites in the NPs-treated samples, regardless of the temperature. Notably, the NPs-treated plants at 35 °C had greater or equal content compared to the NP 25 °C. The expression of total phenols and flavonoids in roots was significantly affected by temperature and NPs treatment, with a much more pronounced effect of NPs (Table S4).

It is noticeable that both in leaves and roots, the bound phenolic compounds and flavonoids had the highest values in the C 35 °C; nonetheless, this contribution did not affect the total metabolites, which were either the lowest (phenolics) or the second lowest (flavonoids) amongst all the samples, due to significantly smaller amounts of the free forms.

## 3. Discussion

### 3.1. Nanoplastics Uptake

A growing body of literature, employing different methodological approaches and techniques [16], has demonstrated that the smallest fractions of microplastics, and especially nanoplastics, can bypass the natural barriers of plant cells, including the cell wall, and enter plant cells [15,16,17,18,19]. These materials can move via apoplastic or symplastic pathways [20]. While endocytosis is considered the main entry route into the protoplast, it has only been observed in isolated cells [21]. At the root level, NMPs must cross the endodermis to reach the vascular system. The primary mechanism involved is the crack-entry mode, which exploits natural discontinuities in the Casparian strip, particularly in the immature endodermis at the root apex and lateral root zones, common entry points for pathogens and nanoplastics alike [22]. This intrusion triggers a range of plant responses and often leads to varying degrees of interference in growth and development, due to cytotoxic and genotoxic effects. However, the impact of nanoplastics on plants under climate change conditions, particularly in the context of rising temperatures, remains largely unexplored.

The use of nanopolystyrene conjugated with a fluorescent tracer enabled us to detect the presence of nanoplastics within various root compartments and to observe both a higher fluorescence intensity and a more widespread distribution pattern when the treatment was conducted at 35 °C. This finding is consistent with our previous study on *Azolla filiculoides* [3], where a similar phenomenon was observed despite the marked differences between the two species, a terrestrial monocot and an aquatic fern.

Rising temperatures may enhance the uptake of nanoplastics in plants through a combination of physiological and biophysical mechanisms. Elevated temperatures increase membrane fluidity, potentially facilitating both passive diffusion and active transport processes such as endocytosis [23]. Additionally, higher temperatures typically stimulate transpiration and water uptake, promoting the mass flow of solutes [24] and potentially nanoplastics, from roots to shoots via the apoplast and xylem. Furthermore, temperature-induced changes in the physicochemical properties of nanoplastics, such as reduced aggregation and altered surface charge, may improve their mobility and bioavailability [25]. Taken together, these findings suggest that, at least within our experimental system, elevated temperatures, such as those anticipated under climate change scenarios, may intensify plant exposure to and internalization of nanoplastics.

### 3.2. Effect of Heat and Nanoplastics on Plant Growth Parameters and Photosynthetic Pigments Content

High temperatures generally negatively affect the growth and development of wheat [26,27]. When they occur during germination and early development, they lead to a reduction in germination rates and seedling growth [6]. Consistent with this, the observed reduction in root and leaf length at higher temperatures reinforces the well-established link between temperature and plant growth. Regarding nanoplastics, the reported effects on plant growth are conflicting, with some studies documenting increases in root and shoot biomass and/or length that depend on the size and concentration of the applied nanoplastics [5,28].

In line with our findings, a low concentration (0.01–10 mg/L) of polystyrene nanoplastics, with a larger size than those used in the present experiment, promoted root and shoot growth in wheat [17]. Although in our work both temperature and nanoplastics significantly affected growth parameters, the high F-value and its statistical significance suggest that temperature plays a predominant role (Table S1). Temperature was also identified as the primary determinant influencing photosynthetic pigments concentrations. Although nanoparticle exposure generally induced an upward trend in pigment content at both temperature regimes, the differences were not statistically significant. The temperature-dependent decrease in chlorophylls and carotenoids concentration aligns with the reduction in pigment content observed at 35 °C in several *T. aestivum* varieties, compared to the control at 25 °C [29]. In the context of a general reduction in pigment levels, the increase in the Chla/Chlb ratio may indicate an attempt to enhance the light-harvesting capacity under the restrictive conditions imposed by high temperatures. However, the enhanced light-harvesting capacity may increase the risk of photoinhibition [30]. Thus, the concurrent rise in the Car/total Chl ratio, especially in NP at 35 °C, likely reflects the plant’s effort to protect its photosynthetic system, given the crucial protective role of carotenoids.

### 3.3. Effects of Heat and Nanoplastics on Oxidative Stress Markers: Hydrogen Peroxide and TBARS Content

As is commonly recognized, both high temperatures [31] and NPs [32] can induce in plants an overproduction of ROS, leading to oxidative stress. In fact, when reactive oxygen species, with hydrogen peroxide playing a central role, accumulate, they can cause injury to macromolecules and cellular structures, resulting in oxidative damage. Indeed, we found a good positive correlation (r = 0.64) between H_2_O_2_ and TBARS levels, suggesting hydrogen peroxide-dependent damage.

Interestingly, leaf H_2_O_2_ levels, consistently higher in the presence of nanoplastics, were lower at elevated temperatures, possibly due to the activation of antioxidant responses under these stressful conditions. This observation aligns with the low levels of TBARS detected at 35 °C. Evidence suggests that elevated temperatures may occasionally attenuate the negative effects of nanoplastics, as observed in a study assessing the combined influence of soil temperature variability and nanoplastic contamination on wheat [10].

Histochemical analysis also revealed clear signs of oxidative stress and damage, not detectable through a biochemical approach, which typically provides an overview at the whole-organ level. Intensely stained regions on the leaf blade as a response to dyes specific for H_2_O_2_ and lipid peroxidation were indeed detectable. These stained areas exhibited a distinct and reproducible spatial pattern that was exclusively associated with nanoplastic exposure, and were consistently observed at both 25 °C and 35 °C. The localization and consistency of these patterns strongly suggest a specific mode of interaction between nanoplastics and plant tissues, potentially indicating targeted sites of stress response or possible accumulation within the leaf structure.

The roots exhibited a different response compared to the leaves, showing little to no variation in the concentrations of the oxidative markers measured. This organ generally exhibited lower levels of oxidative stress compared to the leaves, which is consistent with the higher activity of the antioxidant enzymes APX and POX observed specifically in the roots. Although this result pertains to the entire root, the situation at the root apex appears to be different. Histochemical analyses revealed distinct treatment-dependent differences in this specific region, where the presence of nanoplastics in the growing medium enhanced the reactivity of probes for H_2_O_2_ and lipid peroxidation at both 25 °C and 35 °C. Moreover, oxidative stress markers were more pronounced, both in intensity and diffusion, under NP treatment at 35 °C, suggesting that the root apex is particularly sensitive to these combined stress conditions, as previously reported in rice following nano-PS [19] and nano-ZnO exposure [33].

### 3.4. Effect of Heat and Nanoplastics on the Antioxidant Response

To mitigate oxidative stress, plants have evolved a sophisticated antioxidant defense system comprising both enzymatic components and non-enzymatic molecules. Proline is a compatible solute that accumulates in response to different stress conditions [3,34]. It aids in osmotic adjustment, protects proteins and membranes, and helps in the detoxification of reactive oxygen species [35]. The highest concentration of proline was observed in leaves in C 35 °C, which aligns with the suggested important role of this molecule in protecting against high temperatures [3,36]. Contrary to what was observed in *A. filiculoides* [3], however, the presence of NPs significantly reduced the concentration of proline at the higher temperature. In NP 35 °C, the plant’s ability to manage oxidative stress, as reflected by the relatively low levels of oxidative damage, may be attributed to the concomitant enhanced activity of the antioxidant enzymes APX and POX. Proline levels exhibited distinct patterns in the roots and leaves as temperature fluctuated, reaching their highest values at either low or high temperature, respectively. However, in both cases, the addition of NPs led to a decrease in the levels of this molecule. Consistent with these findings, a depressive effect of 100 nm-sized polystyrene nanoparticles on proline concentration has been demonstrated in cucumber, where it was attributed to disruptions in the metabolism of this osmoprotectant [37]. Protecting proteins is crucial for cell survival under stress conditions [38]. High temperatures can reduce soluble protein content due to denaturation or inhibition of protein synthesis [39]. The observed increase in soluble protein content in the treated roots, along with its stable level in the leaves, suggests that the wheat seedlings are capable of coping with the stressful conditions induced by high temperatures and NPs. Additionally, since soluble proteins can also act as osmotic regulators [40], they may help in osmoregulation when proline levels decrease in the treated seedlings.

Among the antioxidant enzymes, APX plays a crucial role in the AsA-GSH cycle, protecting plants from oxidative stress by converting H_2_O_2_ into water. Its critical function in scavenging H_2_O_2_, particularly within chloroplasts where catalase (CAT) is absent, has been well-documented [41]. Consistent with its important protective role, the highest APX activities in the leaves were observed at 35 °C, with significantly lower values at 25 °C. Similar increases in APX activity at elevated temperatures have been reported in wheat [23], as well as in rice cultivars [42]. At high temperatures, the presence of NPs significantly reduced the activity of this protective enzyme, further highlighting the detrimental effects of the combined stress factors, which exhibited significant interactions with each other. In addition to its role in hydrogen peroxide detoxification, POX is involved in various metabolic processes, such as phenolic metabolism and cell wall lignification, and plays a key role under stressful conditions [43]. The increased activity of APX and POX at 35 °C may compensate for the reduction in catalase activity observed under this treatment. Catalase is a key antioxidant enzyme that directly scavenges H_2_O_2_ through a dismutation reaction [44], and its reduced activity at 35 °C suggests a higher sensitivity to thermal stress compared to APX and POX. Overall, in the leaves, elevated temperatures coupled with the presence of NPs led to a decrease in the activity of the evaluated antioxidant enzymes (APX, POX, and CAT), emphasizing the harmful effects of the combined stress factors. The situation observed in the roots differed from that in the leaves, and the activity of antioxidant enzymes either remained unchanged across treatments (POX), decreased at 35 °C (APX), or was lower in treated plants compared to C 25 °C (CAT). Notably, the activities of POX and APX were higher in the roots than in the leaves, consistent with the lower hydrogen peroxide concentration observed in the root tissues.

### 3.5. Effect of Heat and Nanoplastics on Polyphenols Content

Besides the induction of antioxidant enzymes, part of the plant strategy for coping with abiotic stress relies on the accumulation of secondary metabolites. Polyphenols act as potent antioxidants that counteract ROS, contribute to membrane stability, help maintain osmolyte balance, and are involved in regulating signal transduction pathways associated with stress responses [45]. As a stress response, soft wheat polyphenols accumulate upon moderate and severe heat treatments [45,46], as well as NPs/MPs administration [47,48]. In our experiments, leaf phenolic compound levels consistently declined across all treatments, reaching their lowest values at 35 °C and in the presence of NPs. This decline could be the result of an efficient antioxidant enzyme machinery, as shown by the fluctuations in APX, POX, and CAT activities, thus resulting in a lesser need for polyphenols to counteract oxidative stress. Interestingly, the bound polyphenols had significantly higher values in the 35 °C treatment. The increment of these cell wall-bound molecules could be the result of the synthesis of cell wall material and would be in accordance with the increased POX activity observed at 35 °C. On the other hand, roots showed an increasing trend in total polyphenols when subjected to rising temperature or NPs treatment. Both factors combined determined a further increase in root flavonoids, with a prevalent effect of the NPs treatment as indicated by the high F-value for the interaction of the two factors. This could be an attempt to compensate for the reduced (or unchanged) antioxidant enzyme activities, with an increased investment of the plant towards the secondary metabolism, and would be consistent with the upregulation of genes involved in flavonoid biosynthesis induced in wheat roots upon PE-NPs treatment, as found by Zhuang et al. [48].

## 4. Materials and Methods

### 4.1. Plant Material and Treatments

Caryopses (referred to in this paper as grains) of *T. turgidum* L. ssp. *durum* (Desf.) Husn. cv. Cappelli were obtained from plants cultivated in experimental fields of the University of Pisa, Italy. Grains with full viability (11% moisture content and 100% germination after 48 h of soaking) were surface sterilized in sodium hypochlorite solution containing 1% (*v*/*v*) available chlorine for 3 min, then rinsed prior to use. The sterilized grains were germinated in glass Petri dishes for 48 h in the dark at 25 °C, then randomly transferred into glass pots (12 replicates of 50 grains each, with a volume of 12 mL). These pots were placed in two separate growth chambers maintained at different temperatures: 25 °C and 35 °C, both under identical light/dark cycles (16 h/8 h). The experimental design included control groups consisting of pots containing only deionized water (C 25 °C and C 35 °C), and treatment groups containing an aqueous suspension of green fluorescent polystyrene nanoparticles (NPS, nominal size 30 nm; Fisher Scientific S.A.S., Illkirc, France) at a concentration of 50 mg L^−1^ (NP 25 °C and NP 35 °C). This concentration was selected based on values commonly employed in hydroponic studies and was deliberately chosen to simulate a plausible future contamination scenario, reflecting potential environmental trends associated with increasing plastic pollution [11,12]. Both control and treated seedlings were maintained in the growth chambers for 7 days. At the end of the experiment, seedlings were thoroughly rinsed with deionized water. Root and leaf lengths were measured, and samples were either immediately processed for histochemical analysis or flash-frozen in liquid nitrogen and stored at −80 °C for later biochemical analysis.

### 4.2. In Situ Assessment of Nanoplastics Uptake, and Histochemical Evaluation of Oxidative Stress Markers

Ten roots of comparable size and length were selected at random from seedlings under each treatment. These roots were sectioned using a hand microtome, with cuts made about 3–4 mm from the root tip. Using nano-PS labeled with a green fluorescent dye, we tracked their distribution in root cross-sections. These sections were examined under a fluorescence microscope (Leica DMLB, equipped with appropriate sets of excitation/emission filters and supplied with a Leica DFC7000 T camera, Leica Microsystems, Wetzlar, Germany). For semiquantitative histochemical analysis, at least four images of randomly selected sections from each sample were acquired under identical exposure settings to ensure inter-sample comparability. ImageJ 1.53K version software [49] was used for image analysis to quantify integrated density (in arbitrary units), corresponding to fluorescence intensity.

Histochemical evaluation of oxidative stress markers in root cross-sections was performed employing specific fluorescent probes. Amplex UltraRed Reagent (Life Technologies, Carlsbad, CA, USA) was applied for in situ detection of H_2_O_2_ [50]. After staining, slices were mounted in glycerol and observed with a fluorescence microscope (568 ex/681 em nm). BODIPY 581/591 C11 (Life Technologies, Carlsbad, CA, USA) was used as a fluorescent probe to detect lipid peroxidation, indicated by a shift in fluorescence emission from red to green [19]. Microscopic analysis involved simultaneous acquisition of green (485 ex/510 em nm) and red (581 ex/591 em nm) fluorescence signals, which were then merged to create a composite image.

To prevent interference from chlorophyll’s red autofluorescence, leaf samples were analyzed using specific probes detectable via light microscopy. The in situ detection of hydrogen peroxide in shoots was carried out using 3,3′-diaminobenzidine (DAB) staining, as described by Spanò et al. [51]. DAB reacts with hydrogen peroxide in plant tissues, forming dark brown precipitates. Leaf apices were immersed in a freshly prepared solution containing 1 mg mL^−1^ DAB for 4 h at 25 °C in complete darkness, followed by decolorization in 96% ethanol at 65 °C for 60 min. After thorough rinsing, the samples were immediately examined under a light microscope.

Lipid peroxidation was assessed histochemically using Schiff’s reagent (VWR Chemicals BDH, Radnor, PA, USA), which binds to free aldehyde groups and serves as a qualitative marker of lipid peroxidation [14]. Apical leaf segments were incubated in the dye for 60 min at room temperature, then cleared in 96% ethanol at 65 °C for another 60 min. The appearance of a purple coloration was evaluated under light microscopy.

### 4.3. Extraction and Determination of Photosynthetic Pigments

Chlorophylls (*a*, *b*, and total) and carotenoids in the leaves were extracted and measured following the method described by Spanò and Bottega [34]. In summary, the leaves were ground in 80% acetone, and the mixture was centrifuged at 6000× *g* for 10 min at 4 °C. The resulting supernatants were collected, while the pellets were re-extracted with 80% acetone until they lost all color. All supernatants were combined and analyzed using a spectrophotometer at 645 nm, 663 nm, and 470 nm. Pigment concentrations were calculated and reported in mg g^−1^ FW.

### 4.4. Extraction and Determination of Oxidative Stress Markers: Hydrogen Peroxide and Thiobarbituric Acid Reactive Substances

Leaves and roots were homogenized in 50 mM phosphate buffer at pH 6.5, followed by centrifugation. The resulting supernatant was then reacted with 0.1% titanium chloride in 20% (*v*/*v*) sulfuric acid, and absorbance was measured at 410 nm [52]. Hydrogen peroxide concentrations were quantified using a standard curve and expressed in μmol g^−1^ FW. Lipid peroxidation levels were estimated by measuring thiobarbituric acid reactive substances (TBARS) [53]. Plant samples were homogenized in 5% trichloroacetic acid (TCA), and the extract was mixed with TBA reagent (0.5% TBA in 5% TCA). Absorbance was recorded at 532 nm, with non-specific absorbance at 600 nm subtracted. TBARS concentration was calculated using an extinction coefficient of 155 mM^−1^ cm^−1^ and expressed as nmol g^−1^ FW.

### 4.5. Proline Extraction and Determination

Proline concentration was measured using the method described by Bates et al. [54], with slight modifications according to Spanò et al. [55]. Plant material was homogenized in 3% sulfosalicylic acid, and the homogenate was centrifuged at 6000× *g* for 20 min. The supernatant was then mixed in equal volumes (1:1:1) with glacial acetic acid and acid ninhydrin reagent. The mixture was incubated in a boiling water bath at 100 °C for 60 min. After cooling to room temperature, toluene was added to extract the chromophore. The absorbance of the toluene phase was measured at 520 nm using a spectrophotometer. Proline concentration was calculated using a standard curve and expressed as µmol g^−1^ FW.

### 4.6. Extraction and Determination of Antioxidant Enzyme Activities

Plant samples were extracted in 100 mM potassium phosphate buffer (pH 7.5) containing 1 mM EDTA, 30% polyvinylpyrrolidone (PVP-40), and 0.1 mM phenylmethylsulfonyl fluoride (PMSF), following the procedure of Spanò et al. [55] with slight modifications.

Ascorbate peroxidase (APX; EC 1.11.1.11) activity was determined by measuring the decrease in absorbance at 290 nm due to the oxidation of ascorbate, using an extinction coefficient of 2.8 mM^−1^ cm^−1^ [56]. A blank was included to correct for the non-enzymatic oxidation of ascorbate by hydrogen peroxide. Catalase (CAT; EC 1.11.1.6) activity was assessed according to Aebi [57], and activity was calculated based on the decomposition of hydrogen peroxide at 240 nm, using an extinction coefficient of 39.4 mM^−1^ cm^−1^. A blank containing only the enzyme extract was used for correction. Guaiacol peroxidase (POX; EC 1.11.1.7) activity was evaluated following the method of Arezki et al. [58]. The reaction mixture contained 1% guaiacol, and the rate of guaiacol oxidation by hydrogen peroxide was monitored at 470 nm (extinction coefficient of 26.6 mM^−1^ cm^−1^). One unit of POX activity was defined as the amount of enzyme that oxidizes 1.0 μmol of guaiacol per minute. All enzyme assays were performed at 25 °C, and enzymatic activities were expressed as U mg^−1^ protein. Protein concentration (mg g^−1^ FW) was determined using the Bradford method [59], with bovine serum albumin (BSA) as the standard.

### 4.7. Polyphenols Extraction and Quantification

Leaf or rootlet samples subjected to the various treatments were extracted with 80% methanol in water at 5 °C. The supernatant obtained after centrifugation was further treated to obtain the free phenolics, while the pellet was extracted for the bound phenolics, following Fontanini et al. [60]. Briefly, after evaporation of the methanol, the water residue was used for free phenols and flavonoids quantification (see below), whereas the pellet was dried, subjected to alkaline hydrolysis and extracted by partitioning with diethyl ether/ethyl acetate (*v*/*v*, 1:1); the resulting organic phase was dried and the powder obtained was suspended in water and used for the quantification of bound phenolic compounds and flavonoids.

The phenolics assay was a slight modification [59] of the Folin–Ciocalteu method [61]. The reaction product absorbance was read at 760 nm, against a blank where the phenolic extract was replaced by water. Phenolic compounds were quantified based on a calibration curve made with gallic acid (Sigma-Aldrich, Milan, Italy) and the results expressed as mg GAE g^−1^ FW.

The flavonoid assay was based on the colorimetric method described by Dewanto et al. [62]. The solution absorbance was measured at 510 nm against a blank where water replaced the sample. Flavonoids were quantified based on a calibration curve made with catechin (Sigma-Aldrich, Milan, Italy), and the results were expressed as mg CE g^−1^ FW. Each assay had three replicates.

### 4.8. Statistical Analysis

All results were repeated at least four times. Data are reported as the mean ± SE. Statistical analyses were performed using two-way ANOVA, followed by a Tukey post hoc test, and different letters denote statistical significance at *p* < 0.05. Interaction terms and their associated *p*-values are shown in Tables S1–S5.

## 5. Conclusions

This study provides novel insights into the combined effects of nanoplastics and elevated temperature on durum wheat physiology, highlighting complex and tissue-specific responses. Our findings demonstrate for the first time that high temperatures can enhance the uptake and preferential accumulation of nanoplastics in crops, suggesting a potential interaction between thermal stress and nanoparticle internalization and distribution. This accumulation correlates with intensified oxidative stress in the root apex, a critical zone for plant development. Notably, while nanoplastics alone disrupt redox balance and reduce proline levels, heat stress induces proline accumulation and compromises growth with limited activation of secondary defenses. Under combined stress, symptoms are exacerbated: antioxidant defenses are markedly reduced in leaves, whereas roots show a compensatory increase in phenolic compounds. These findings, obtained under hydroponic conditions, raise new concerns about the vulnerability of major crops like wheat to emerging contaminants under climate change scenarios and underline the importance of considering combined abiotic stressors in future risk assessments.

## Figures and Tables

**Table 1:** Leaf length (mm), content of proline (μmol g^−1^ FW), protein (mg g^−1^ FW), and activity (U mg^−1^ protein) of ascorbate peroxidase (APX), guaiacol peroxidase (POX), and catalase (CAT) in leaves of *Triticum turgidum* plants grown at 25 °C or 35 °C in water (C 25 °C, C 35 °C) or in the presence of polystyrene nanoplastics (NP 25 °C, NP 35 °C). Data are reported as mean ± standard error of at least four replicates. Different letters indicate significant differences at *p* < 0.05.

	Leaf Length	Proline	Protein	APX	POX	CAT
C 25 °C	172.78 ± 4.38 b	4.08 ± 0.09 b	3.01 ± 0.21 a	2.09 ± 0.02 d	1.87 ± 0.03 c	4.58 ± 0.31 a
NP 25 °C	191.12 ± 4.03 a	3.98 ± 0.09 b	3.23 ± 0.20 a	2.58 ± 0.04 c	1.96 ± 0.04 c	2.09 ± 0.14 c
C 35 °C	66.20 ± 1.89 d	6.03 ± 0.25 a	3.08 ± 0.09 a	3.50 ± 0.05 a	2.92 ± 0.06 a	3.42 ± 0.23 b
NP 35 °C	85.60 ± 1.86 c	3.65 ± 0.21 b	3.34 ± 0.19 a	2.83 ± 0.05 b	2.43 ± 0.08 b	2.22 ± 0.08 c

**Table 2:** Root length (mm), content of proline (μmol g^−1^ FW), protein (mg g^−1^ FW), and activity (U mg^−1^ protein) of ascorbate peroxidase (APX), guaiacol peroxidase (POX), and catalase (CAT) in roots of *Triticum turgidum* plants grown at 25 °C or 35 °C in water (C 25 °C, C 35 °C) or in the presence of polystyrene nanoplastics (NP 25 °C, NP 35 °C). Data are reported as mean ± standard error of at least four replicates. Different letters indicate significant differences at *p* < 0.05.

	Root Length	Proline	Protein	APX	POX	CAT
C 25 °C	83.78 ± 3.17 b	4.64 ± 0.07 a	1.72 ± 0.03 c	4.78 ± 0.23 a	5.35 ± 0.80 a	3.51 ± 0.17 a
NP 25 °C	97.52 ± 3.12 a	2.92 ± 0.22 b	2.06 ± 0.20 bc	4.89 ± 0.07 a	6.14 ± 0.18 a	2.37 ± 0.18 b
C 35 °C	20.26 ± 1.11 c	3.22 ± 0.12 b	2.79 ± 0.12 a	3.22 ± 0.10 b	5.51 ± 0.34 a	2.45 ± 0.24 b
NP 35 °C	22.46 ± 0.94 c	3.09 ± 0.18 b	2.54 ± 0.11 ab	5.33 ± 0.27 a	5.97 ± 0.44 a	2.82 ± 0.17 ab

**Table 3:** Content (mg g^−1^ FW) of total chlorophyll (Total Chl), carotenoids, chlorophyll *a*/chlorophyll *b* ratio (Chla/Chlb), and carotenoids/total chlorophyll ratio in leaves of *Triticum turgidum* plants grown at 25 °C or 35 °C in water (C 25 °C, C 35 °C) or in the presence of polystyrene nanoplastics (NP 25 °C, NP 35 °C). Data are reported as mean ± standard error of at least four replicates. Different letters indicate significant differences at *p* < 0.05.

	Total Chl	Carotenoids	Chla/Chlb	Carotenoids/Total Chl
C 25 °C	1.16 ± 0.23 a	0.16 ± 0.03 ab	2.71 ± 0.07 a	0.14 ± 0.00 c
NP 25 °C	1.52 ± 0.23 a	0.22 ± 0.03 a	2.65 ± 0.06 a	0.15 ± 0.00 c
C 35 °C	0.32 ± 0.04 b	0.06 ± 0.01 c	1.56 ± 0.10 b	0.21 ± 0.02 b
NP 35 °C	0.44 ± 0.05 b	0.11 ± 0.01 bc	2.03 ± 0.13 b	0.27 ± 0.01 a

## Data Availability

The original contributions are included in the article and Supplementary Files. Further queries can be directed to the corresponding author.

## Supplementary Materials

The following supporting information can be downloaded at https://www.mdpi.com/article/10.3390/plants14152426/s1, Table S1: Two-way ANOVA analysis showing effects of temperature and polystyrene nanoplastics and their interaction on growth and photosynthetic parameters. Table S2: Two-way ANOVA analysis showing effects of temperature and polystyrene nanoplastics and their interaction on the concentration of leaf biochemical parameters. Table S3: Two-way ANOVA analysis showing effects of temperature and polystyrene nanoplastics and their interaction on leaf protein concentration and antioxidant enzyme activities. Table S4: Two-way ANOVA analysis showing effects of temperature and polystyrene nanoplastics and their interaction on the concentration of root biochemical parameters. Table S5: Two-way ANOVA analysis showing effects of temperature and polystyrene nanoplastics and their interaction on root protein concentration and antioxidant enzyme activities.
