# Supplementary material for: Interacting Effects of Heat and Nanoplastics Affect Wheat (Triticum turgidum L.) Seedling Growth and Physiology
Source: Plants (Basel). 2025 Aug 5;14(15):2426. doi: 10.3390/plants14152426 (PMC12349157; doi:10.3390/plants14152426)
Supplement: Supplementary file 1 [file plants-14-02426-s001.zip › plants-3740855-supplementary.pdf]

Table S1. Two-way ANOVA analysis showing effects of temperature and polystyrene nanoplastics and their interaction on growth and photosynthetic parameters.

	Leaves length			Roots length			Total Chlorophyll			Carotenoids			Chla/Chlb			Carot/ Tot Chl		
	df	*F*	*p*	*df*	*F*	*p*	*df*	*F*	*p*	*df*	*F*	*p*	*df*	*F*	*p*	*df*	*F*	*p*
Temperature	1	1025	<0.001	1	876.8	<0.001	1	74.02	<0.001	1	44.01	<0.001	1	37.18	<0.001	1	75.52	<0.001
Nanoplastic	1	32.51	<0.001	1	11.6	<0.001	1	2.189	0.1628	1	8.011	0.014	1	1.265	0.2811	1	11.61	0.0047
Interaction	1	0.1107	0.7397	1	6.08	0.0145	1	1.272	0.2797	1	0.2412	0.6315	1	3.094	0.1021	1	6.899	0.0209

Table S2. Two-way ANOVA analysis showing effects of temperature and polystyrene nanoplastics and their interaction on the concentration of leaf biochemical parameters.

	Hydrogen peroxide			TBARS			Proline			Total Phenols			Total Flavonoids		
	df	*F*	*p*	*df*	*F*	*p*	*df*	*F*	*p*	*df*	*F*	*p*	*df*	*F*	*p*
Temperature	1	35.02	<0.001	1	2504	<0.001	1	36.7	<0.001	1	370.7	<0.001	1	138.2	<0.001
Nanoplastic	1	76.95	<0.001	1	34.98	<0.001	1	85.7	<0.001	1	52.03	<0.001	1	870.1	<0.001
Interaction	1	8.991	0.01499	1	69.48	<0.001	1	72.89	<0.001	1	56.7	<0.001	1	247.2	<0.001

Table S3. Two-way ANOVA analysis showing effects of temperature and polystyrene nanoplastics and their interaction on leaf protein concentration and antioxidant enzyme activities.

	Proteins			APX			POX			CAT		
	df	*F*	*p*	*df*	*F*	*p*	*df*	*F*	*p*	*df*	*F*	*p*
Temperature	1	0.3585	0.5605	1	394.7	<0.001	1	176.3	<0.001	1	12.13	0.0019
Nanoplastic	1	2.311	0.1544	1	4.812	0.0487	1	12.37	0.00425	1	112.9	<0.001
Interaction	1	0.0188	0.8931	1	189.3	<0.001	1	25.83	<0.001	1	12.14	0.0019

Table S4. Two-way ANOVA analysis showing effects of temperature and polystyrene nanoplastics and their interaction on the concentration of root biochemical parameters.

	Hydrogen peroxide			TBARS			Proline			Total Phenols			Total Flavonoids		
	df	*F*	*p*	*df*	*F*	*p*	*df*	*F*	*p*	*df*	*F*	*p*	*df*	*F*	*p*
Temperature	1	0.0815	0.7825	1	1.87	0.1965	1	370.7	<0.001	1	192.8	<0.001	1	1552	<0.001
Nanoplastic	1	9.645	0.0145	1	3.719	0.0777	1	52.03	<0.001	1	1329	<0.001	1	3286	<0.001
Interaction	1	0.2768	0.6131	1	73.43	<0.001	1	56.7	<0.001	1	316	<0.001	1	972.2	<0.001

Table S5. Two-way ANOVA analysis showing effects of temperature and polystyrene nanoplastics and their interaction on root protein concentration and antioxidant enzyme activities.

	Proteins			APX			POX			CAT		
	df	*F*	*p*	*df*	*F*	*p*	*df*	*F*	*p*	*df*	*F*	*p*
Temperature	1	35.22	<0.001	1	4.837	0.0465	1	<0.001	0.9953	1	1.418	0.2427
Nanoplastic	1	0.1045	0.7521	1	31.62	<0.001	1	1.404	0.2558	1	2.814	0.1035
Interaction	1	5.133	0.04278	1	24.4	<0.001	1	0.0569	0.8149	1	16.58	<0.001
